# Contributions to the Study of the Physical Diagnosis of Diseases of the Chest

**Published:** 1850-07

**Authors:** Austin Flint

**Affiliations:** Buffalo


ART. III.—*Contributions to the Study of the Physical Diagnosis of Diseases of the Chest.* By Austin Flint, M. D.

The following observations, developed by the study of thoracic diagnosis at the bedside, the writer has ventured to think are not without some interest and practical value, as well as originality, and they are therefore respectfully submitted to those engaged in cultivating the art of discriminating pulmonary diseases by means of physical signs.

*Determination of dullness of resonance by the pitch, or note elicited by percussion.*—I have derived, of late, much assistance in determining the existence of dullness on percussion on one side of the chest, compared with the other side, by directing attention to the character of the *note* as respects *elevation*, or *pitch*. The usual method with those who practice physical exploration, in so far as the acquaintance of the writer extends, is to compare the two sides with reference to a disparity of resonance, and to estimate the degree of disparity, without observing if there be a deviation in harmony of the sounds considered as musical notes. If there be absolute flatness, or an amount of dullness approximating thereto, it is by no means difficult to determine the fact, even if the explorer be inexperienced. Under these circumstances, a comparison of the two sides is hardly necessary. But when the deviation from normal resonance is less marked, its appreciation is not so easy, and to those who have had but little practice, and are deficient in experience, it is often a difficult problem. In the latter case as is well known, we do not estimate dullness by any abstract, or average standard—this cannot be done, inasmuch as the chests of different persons differ considerably in healthy resonance, owing to various causes—but we judge by comparison of one side with the other, assuming, as we may do, that in the vast proportion of cases, the two sides emit, on percussion, (excepting the præcordial region,) an equal resonance. The question in cases of doubt is, are the two sides, at the various points equidistant from the mesial line, equally resonant? Attention to the *pitch* or *elevation of the note*, will, in every case, obviate all doubt as to the answer to this question, provided the explorer have a correct ear, and especially if he has ever cultivated the ability to discriminate nicely musical sounds. *If a disparity exist, the duller resonance invariably has a higher elevation, or pitch;* in other words, the dull sound has a greater number of vibrations. A striking difference may be at once noticed, in this respect, when it might be difficult to appreciate a disparity of mere resonance without directing attention to a comparison of the notes. This method of determining equality, or difference, is not only accurate, but is much more easy, and is at once acquired by the beginner. I have, on several occasions, tested the facility with which a discrimination may be made by a person wholly unaccustomed to physical diagnosis. Thus—a patient presenting obvious, but not strongly marked relative dullness on one side, from tuberculous deposit, is made the subject of physical examination. The unpracticed ear of an intelligent student, or practitioner, often fails to detect any difference in sound, so long as his attention is directed to the mere resonance. After confessing his inability to perceive any disparity, let him be instructed to listen to the sound produced on each side in succession, as if they were musical notes, and to mark the pitch or elevation. Then, if he have a musical ear, all doubt and difficulty will vanish, and he will feel immediately competent to decide on even a slight variation in the two sides. His delicacy of discrimination will be greater the more he is qualified or accustomed to discover slight shades of musical discords.

To the experienced auscultator there may be nothing new in the foregoing. The subject first suggested itself to the writer at the examination of a patient several months ago. On referring, at that time, to several treatises on physical exploration, he was unable to find any direction attaching to it that importance which it then appeared to him to possess, and which his experience has since confirmed. A difference in pitch, is stated by Bowditch, and Hughes, to be present in some cases of incipient tuberculosis, as a shade of disparity falling somewhat short of positive dullness. It is not, however, mentioned by these, or other writers that have been consulted, as offering a ready and precise means of determining moderate or slight dullness in all cases.

At first I had flattered myself that a discovery had been made by which the proportionate degrees of deviation from normal resonance might be measured—that the scale of gradations in sound would afford a criterion to estimate the amount of disease more accurately than by a rough comparison of resonance—in short, that the *gamut* would be found more minutely applicable to thoracic diagnosis, than in the mere fact of disparity of pitch. Expectations of this kind were, however, soon abandoned, and the value of the idea limited to the objects which have been explained in the foregoing remarks. As assisting to determine quickly and certainly the existence of relative dullness, and as affording a key to the immediate practice of percussion with success by the novitiate, the writer believes it will be found to merit consideration.

The applicability of the foregoing principle may be demonstrated in the healthy chest by comparing the pitch of the sound on percussion over the præcordial region, with that on the corresponding locality on the right chest. The difference in resonance occasioned by the presence of the heart, overlapped by the lung, is not readily appreciable by the unpracticed, except the attention be directed to the note.

*Tympanic resonance, in solidification (from inflammation) of the upper lobe of the lung; and in pleuritis with large effusion.*—Tympanitic resonance associated with bronchial respiration, is a combination of signs which the auscultator would not expect to find in a case of pneumonitis. That such a combination may exist is important to be known, and I do not recollect having seen the fact noticed by writers on the subjeet.

The following case affords an illustration:—

John Donaldson, Englishman, married, draper, aged 40, entered Hospital January 26, 1850. He had lately arrived in this country, and had drank very freely since his arrival, at the same time taking but little food. He had arrived at this city four or five days prior to his entrance, and had been attacked in the railway cars with acute pain in the left chest On his arrival at Buffalo he was visited by Drs. Wallis and Wilcox, and treated by bleeding and vesication. The day after his arrival he became delirious, insisting upon changing his lodgings and medical attendants, refusing to take remedies, and, at times, he was violent. The treatment, following the bleeding and vesication, consisted of antimony, mercury, and opium. He slept the night before entering the hospital, and was more quiet on the day of his entrance than previously, taking remedies readily, but still manifesting delirium by a desire to change lodgings, and incoherent conversation. During the night after his entrance, his delirium was active—he constantly attempted to get out of bed, talking to himself, imagining he was on board vessel, etc.

On the 27th, his symptoms were as follows:

Expression unsettled. Quiet and docile, but continues to talk deliriously, laboring under the delusion that he was treated badly before coming to the hospital. He appears satisfied with his present situation. Respirations 24, and somewhat labored. No dilatation of *alæ nasi*, nose swelled and reddened. Pulse, 136, developed, and thrilling. Skin mellow, and temperature moderately elevated. One dejection. Tongue moist, and clean, and somewhat reddened.

Physical examination:—Flatness on percussion over middle and inferior thirds of left chest, anteriorly and posteriorly. *Resonance at upper third relatively clearer than on the right side—tympanitic in character.* Respiration absent in middle and lower thirds of left chest. At upper third, anteriorly, feeble, bronchial, prolonged expiration; posteriorly, less feeble, bronchial.

The expectoration was small in quantity, and partially opaque.

The patient died during the following night, and the chest was examined the subseqent day. The left pleural cavity contained considerable turbid fluid, with fibrinous flakes. Lower lobe compressed into a small space. Upper lobe but little, if at all contracted, completely solidified, presenting the physical characters of pneumonitis in the second stage. No tubercular deposit. A small layer of fibrin effused on pleural surface of lung.

Right lung presented old pleuritic adhesions. Substance somewhat œdematous.

How is the existence of tympanitic resonance over solidified lung, in this instance, to be explained? The supposition of emphysema of the upper lobe, which suggested itself as the most plausible explanation at the time of the examination, was disproved by the appearances found after death. No evidence of the evolution of gas in the pleural cavity appeared at the examination, and the effused fluid presented no fœtor, or other indications of decomposition.

The only obvious alternative remaining, is to attribute the resonance to the accumulation of gas within the stomach, the sound being conducted by the heart and solidified lung, but not transmitted through the fluid accumulated in the pleural sac, so as to be apparent by percussing the middle and lower regions of the chest.

The same explanation is probably applicable to the following instance, in which a combination of signs, in some respects similar, was presented. In giving this case, details not relevant to the present purpose will be omitted.

James Kelly, Irish, aged 18, had been in hospital several months, as a surgical patient, for chronic, indolent ulcers, which were healed at the time of the present attack. He was transferred to the medical department, 23d January, 1850. A week previously he had been seized with severe, sharp, lancinating pain in the left chest, shooting into the shoulder. The pain continued the following day, but was less severe. The next night he began to cough. The cough was dry, hacking, and attended by pain and soreness in the upper part of the left chest. It was unattended by expectoration. He had high febrile movement, hot skin, furred tongue, anorexia, and thirst. Expectoration continued slight. The details of the history and treatment for the first week, before he came under my notice, are not recorded; nor for the first two days after he was transferred. On the 25th Jan. the physical signs noted, were as follows:—*On percussion, the upper and middle thirds of the left chest yield a much clearer resonance than the corresponding regions of the right side. The resonance has a tympanitic character; over the lower third of the left chest, the resonance tympanitic. Laterally, in the left side, resonance tympanitic; on left shoulder also*

*tympanitic.* Over the right side, resonance normal. On auscultation. in left side anteriorly, respiration feeble, tubular, expiration of same length as inspiration. Posteriorly, respiration feeble, tubular, at upper third; *lower third and part of middle third, no respiration, and flatness on percussion.* Over right side respiration vesicular, supplementary, and expiration somewhat prolonged.

The respirations are 28, slightly labored, without dilatation of alæ nasi. He does not complain of dyspnœa. Pulse 100, and rather small. Tongue moist, and slightly furred. Bowels moved night before last. Anorexia, sweat profusely last night. He is not greatly prostrated. No pain in chest.

Jan. 26th.—Manifest disparity in the two sides, as regards the intercostal depressions. They are marked on the right side, and absent on the left. Mobility of right side slightly greater than of left. Other physical signs the same as on the 25th.

27th.—Aspect improved. The resonance at the upper third of the left chest is less tympanitic, but still clearer than on the right side. Respiration more developed, and approximating to vesicular in character. Pulse 104. Respirations 28, not labored. Expectoration small in quantity. Two sputa observed, solid, of yellowish color, and streaked with blood.

30th.—Improving. Febrile movement continues, but is less in degree. Perspires at night.

Less disparity of resonance at the summit of the chest; but there is still exaggerated clearness on the left side. Tympanitic resonance below line of nipple. Complained of some pain in chest last night. The left chest at inferior lateral portion appears slightly contracted. The two sides were carefully measured several days ago, and found to be exactly equal.

Feb. 13.—Kelly has been constantly improving since the date of the last record. He now sits up most of the day. Tongue clean. Appetite good. Bowels regular. Pulse 100. The chest on the left side, inferiorly, presents distinct contraction, and on measurement is three-fourths of an inch less in circumference than the right. The mobility of the right chest is obviously greater than that of the left. Greater relative clearness of resonance at the upper third of the left chest continues. *Flatness below the level of the nipple.* Respiration at upper third of left chest feeble, and relative duration of expiration increased. Respiration on right side normal. The impulse of the heart is not appreciable, but a slight pulsation can be seen on either side, near the sternum; on the right side, situated above the level of the nipple, on the left, on a level with the nipple. He has no cough, nor expectoration.

Feb. 24th.—Inferior, anterior portion of chest, on left side, manifestly flattened. Left interscapular space diminished. Less expansibility of the left side than of the right. Impulse of heart felt distinctly, on a level with the nipple, between the nipple and the left margin of the sternum. Left chest resonant throughout, but relatively dull. Respiratory murmur feeble over left side, anteriorly and posteriorly.

March 3d.—Improving. Sits up the greater part of the time. Has occasionally sharp pain in chest. Appetite good. Bowels regular. No cough. Pulse somewhat accelerated. He continued to improve, and was quite well at the expiration of my period of service at the hospital, April 1st.

This case interested me much from the constancy of a tympanitic resonance so general and considerable, over the affected side, as, at one time to lead to the suspicion of pneumo-hydro-thorax. In referring to the notes of the case, of which the foregoing history is an epitome, omitting entirely the treatment, and other particulars, I regret to find the account of the physical explorations not so full as I could have desired. I have omitted, in the sketch that has been given, to mention, what is embraced in the records, that the epigastrium emitted a tympanitic resonance on percussion.

It is sufficiently clear that this was a case of pleuritis with large effusion. Assuming that the tympanitic resonance over the chest was due to the presence of gas in the stomach, it was transmitted through the fluid contents of the pleural cavity in every direction, in this respect differing from the case previously given. It will, however, be observed that as the quantity of fluid diminished, as evidenced by the contraction of the chest, the tympanitic sound was present at the upper, but absent at the lower portion of chest, showing, as in the former case, that the resonance was transmitted with greater facility through condensed lung, (condensed in this instance from compression,) than through a collection of liquid. It was interesting, in this case, to watch the development of the normal vesicular resonance, gradually supplanting the tympanitic.

If the rationale we have offered of the peculiar feature of the above cases be correct, the practical inference is, that we may expect, in some instances of pneumonitis, and pleuritis, to find an abnormally clear resonance on percussion, co-existing with the auscultatory phenomena denoting these affections; and, without being aware of this unusual combination of physical signs, the diagnosis may be attended with difficulty.

*Permanent disparity of the resonance, and respiratory sound of the two sides of the chest, the effect of pleuritis and pneumonitis.*—In employing physical exploration, it is important to bear in mind that a marked disparity in resonance, and respiratory sound, may be due to an attack of pleuritis, or of pneumonitis, experienced many years prior to the examination, and, hence, is not to be considered, necessarily, evidence of present disease. Equality in resonance and the respiratory murmur obtains in the vast majority of persons in whom the thoracic organs are healthy, and this rule is of fundamental importance in deriving information from physical signs. A slight disparity, taken in connection with symptoms, and history, often suffices to decide the question as to the existence of tuberculous deposit in the lungs. It is in suspected cases of pulmo-tuberculosis that the rule just mentioned is of especial consequence. If a patient have severe pneumonitis, and recover complete health, the affected lung, nevertheless, may never regain the same amount of vesicular expansion that it had before the attack. The vesicular structure is not compromised in a degree to occasion any evidences that the pulmonary function is impaired, for, in the conservative arrangements of Providence, a capacity of lungs is provided beyond that which the necessities of the organism require. The patient may not be in the least conscious of any defect, while an injury has been produced sufficient to disturb the natural harmony existing between the two sides of the chest. The same effect may follow an accumulation of liquid in the pleural sac, which is completely absorbed, leaving the chest more or less contracted on one side, and the pleural membrane thickened by the organization of the effused fibrin. In either case the proportion of air to solids is lessened, and, hence, there is, ever after, more or less relative dullness on percussion, and a relatively feeble respiratory murmur. On this account it is not so easy to determine, in all cases, the existence of tuberculous deposit, if the patient have, at any past period of his life, suffered from pleuritic, or pneumonic inflammation, as it usually is, if these diseases have not preceded. The importance of obtaining information on this point in cases which present themselves for examination, is, at once, obvious. I have several times met with instances in which a difficulty in diagnosis proceeded from this cause. The following case, which came under observation but a few days since, will serve as an illustration:—A patient over forty years of age, applied to be examined for life assurance. No evidence appeared from his history, parentage, or present symptoms, of tuberculous disease, unless the existence of chronic pharyngitis, of the variety called granular, be so considered, which, in so far as the observations of the writer go, indicates the reverse of a tubulous cachexy. On exploration of the chest, the right side presented a slight dullness of resonance compared with the left side, and there existed, on the same side, a relatively feeble respiratory murmur. Under these circumstances, tuberculosis was suspected, notwithstanding the patient supposed himself to be well, save the pharyngitis, his aspect denoting health, and nothing, as just remarked, sustaining the suspicion of phthisis derived from the disparity between the two sides of the chest. The patient had never been seriously ill but once, and in that instance he had an attack of fever, followed by inflammation of the right lung. That the right lung was the one affected was shown by the marks of cupping on that side, which were still apparent. This was eighteen years previous. The probability is, that in this instance there is no present disease of the lungs; but the permanent effects of a severe pneumonitis, experienced so many years before, will always render it somewhat more difficult to appreciate the early physical signs of tuberculosis, than if he had never been the subject of pneumonic inflammation.

*New method of demonstrating the presence of liquid effusion into the cavity of the pleura on one side.*—The physical signs of an accumulation of liquid in the pleural sac are, already, sufficiently ample and explicit; the following additional method, however, may possess some practical value: Suppose a case of pleuritic effusion, limited to one side. If pressure be made on the healthy side, upon the cartilages of the false ribs, nigh to the sternum, an impulse is communicated to the opposite side, which is seen to be communicated equally to the *whole* of the affected side. If, then, an equal pressure be made over a corresponding point on the affected side, i. e. upon the cartilages of the false ribs, nigh to the sternum, the healthy side will be elevated nigh to the sternum, but the whole chest will exhibit little or no motion. The difference in the two instances will be obvious.

The philosophy of the difference is supposed to be this:—when pressure is made on the healthy side, over the yielding cartilages nigh to the sternum, the impulse is communicated to the mediastinum, thence to the liquid contents of the pleural sac, and, by a well known law of hydrostatics, the momentum is distributed by the latter, equally, in all directions. This accounts for the motion being communicated to the whole side, in that instance. When, on the other hand, the pressure is made on the affected side, in the same relative situation, the impulse, in like manner communicated to the mediastinum, is transmitted to the lung, which readily yields, causing an elevation of the chest directly above it, i. e. nigh to the sternum; but the momentum being lost in the elasticity of the lung, motion is not transmitted to the whole side, or in a less degree than when pressure is made on the healthy chest, in the former instance.

By this method the presence of liquid effusion in one side is shown. The writer has not made the experiment of a similar comparison of pressure on the two sides, in a case of solidification of the lower lobe of the lung on one side.

BUFFALO, May, 1850.

